# Supplementary material for: Human dynein–dynactin is a fast processive motor in living cells
Source: eLife. 2026 Mar 25;13:RP94963. doi: 10.7554/eLife.94963 (PMC13016606; doi:10.7554/eLife.94963)
Supplement: Figure 3—source data 1. [file elife-94963-fig3-data1.pptx]

## Slide 1
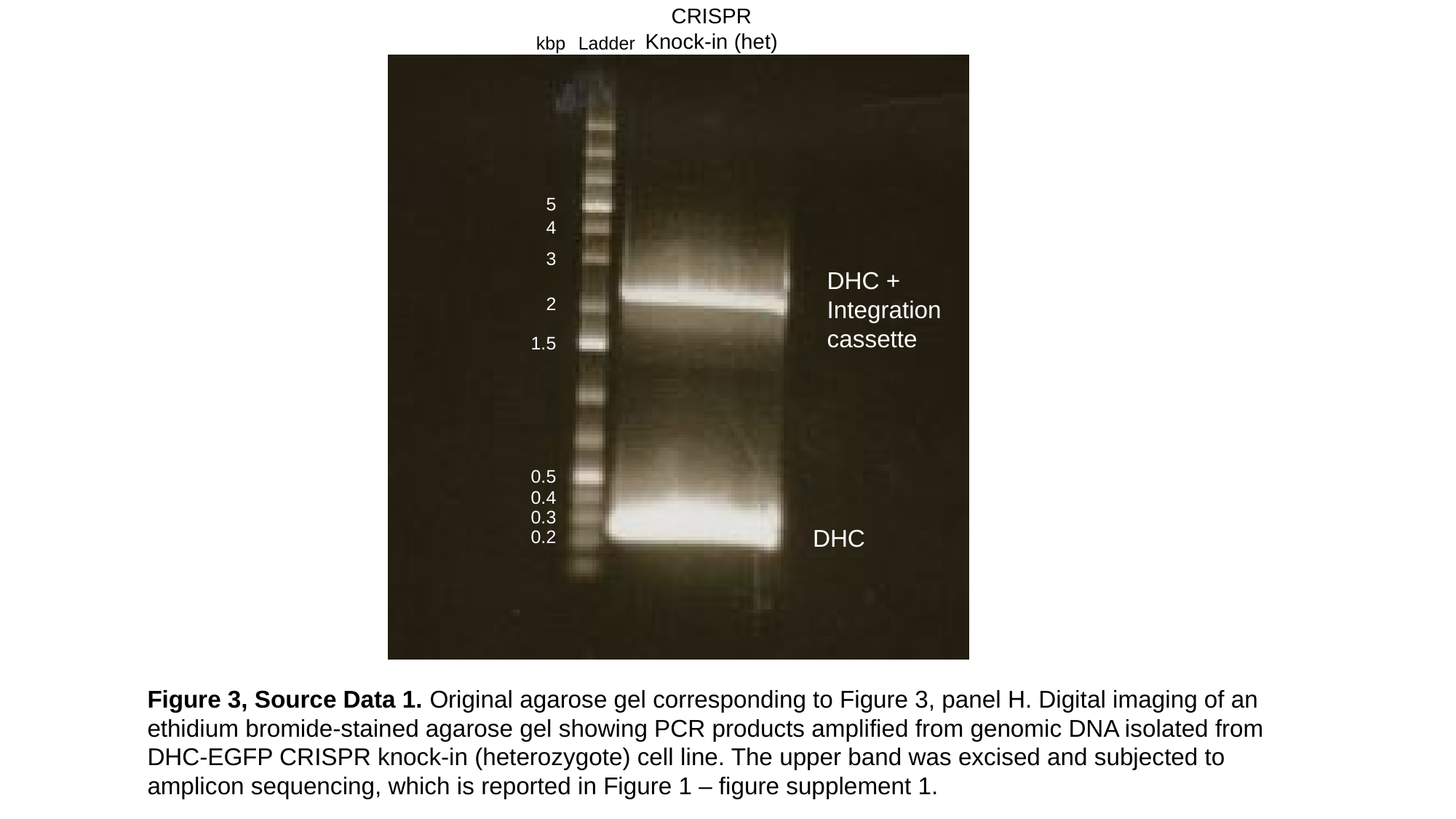

CRISPR
Knock-in (het)
kbp
Ladder
5
4
3
DHC +
Integration
cassette
2
1.5
0.5
0.4
0.3
DHC
0.2
Figure 3, Source Data 1. Original agarose gel corresponding to Figure 3, panel H. Digital imaging of an ethidium bromide-stained agarose gel showing PCR products amplified from genomic DNA isolated from DHC-EGFP CRISPR knock-in (heterozygote) cell line. The upper band was excised and subjected to amplicon sequencing, which is reported in Figure 1 – figure supplement 1.
