# Supplementary figures and images for: Human dynein–dynactin is a fast processive motor in living cells
Source: eLife. 2026 Mar 25;13:RP94963. doi: 10.7554/eLife.94963 (PMC13016606; doi:10.7554/eLife.94963)

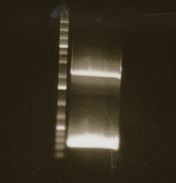

Supplement: Figure 3—source data 2. [file elife-94963-fig3-data2.zip › Figure3ΓÇöSourceData2/Dynein_Het_PCR (2023_02_19 17_55_48 UTC).JPG]

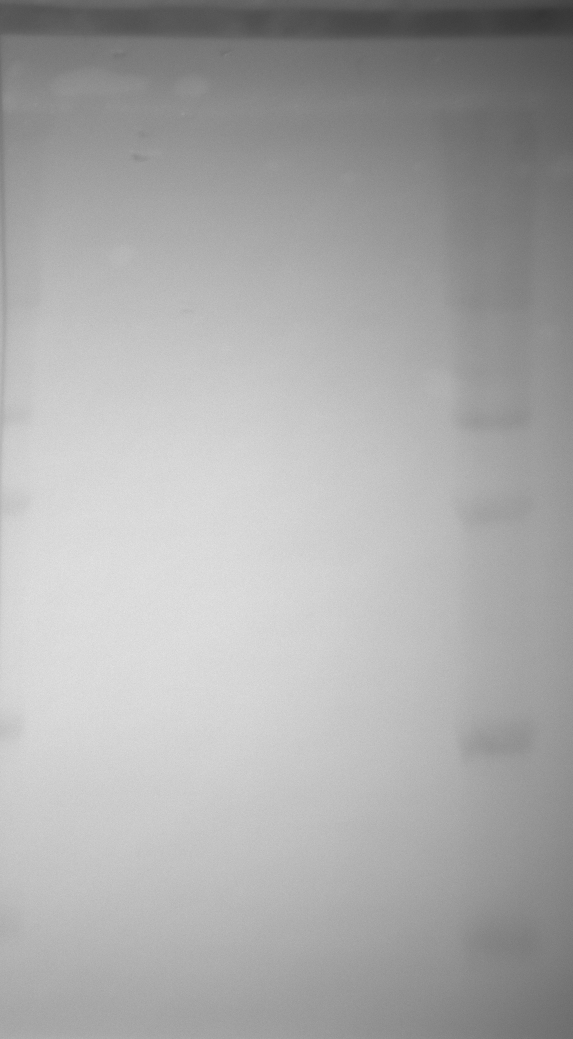

Supplement: Figure 3—source data 4. [file elife-94963-fig3-data4.zip › Figure3ΓÇöSourceData4/membrane.tif]

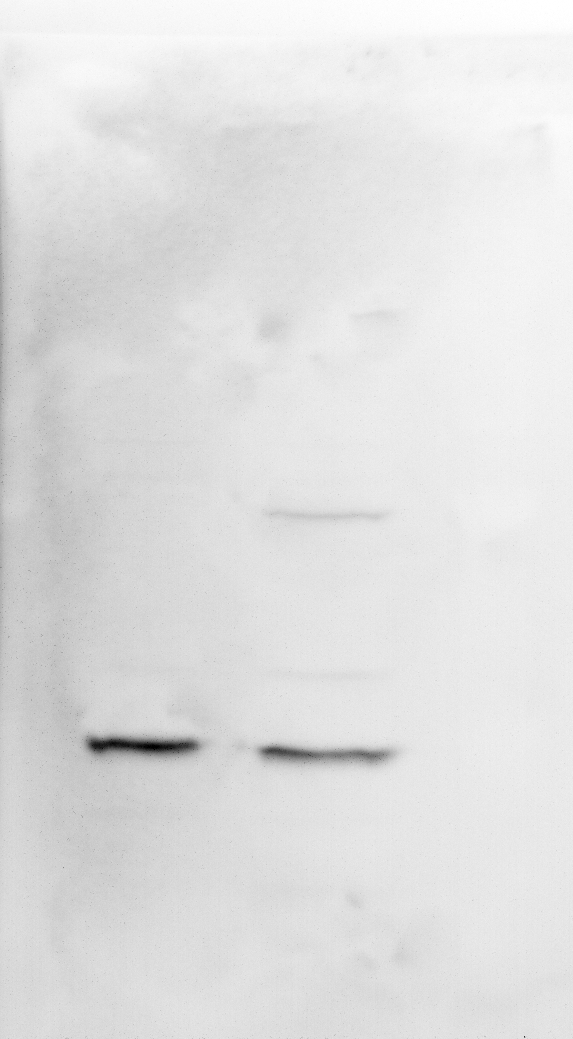

Supplement: Figure 3—source data 4. [file elife-94963-fig3-data4.zip › Figure3ΓÇöSourceData4/p50_blot.tif]
