# Supplementary material for: Human dynein–dynactin is a fast processive motor in living cells
Source: eLife. 2026 Mar 25;13:RP94963. doi: 10.7554/eLife.94963 (PMC13016606; doi:10.7554/eLife.94963)
Supplement: Figure 3—source data 3. [file elife-94963-fig3-data3.pptx]

## Slide 1
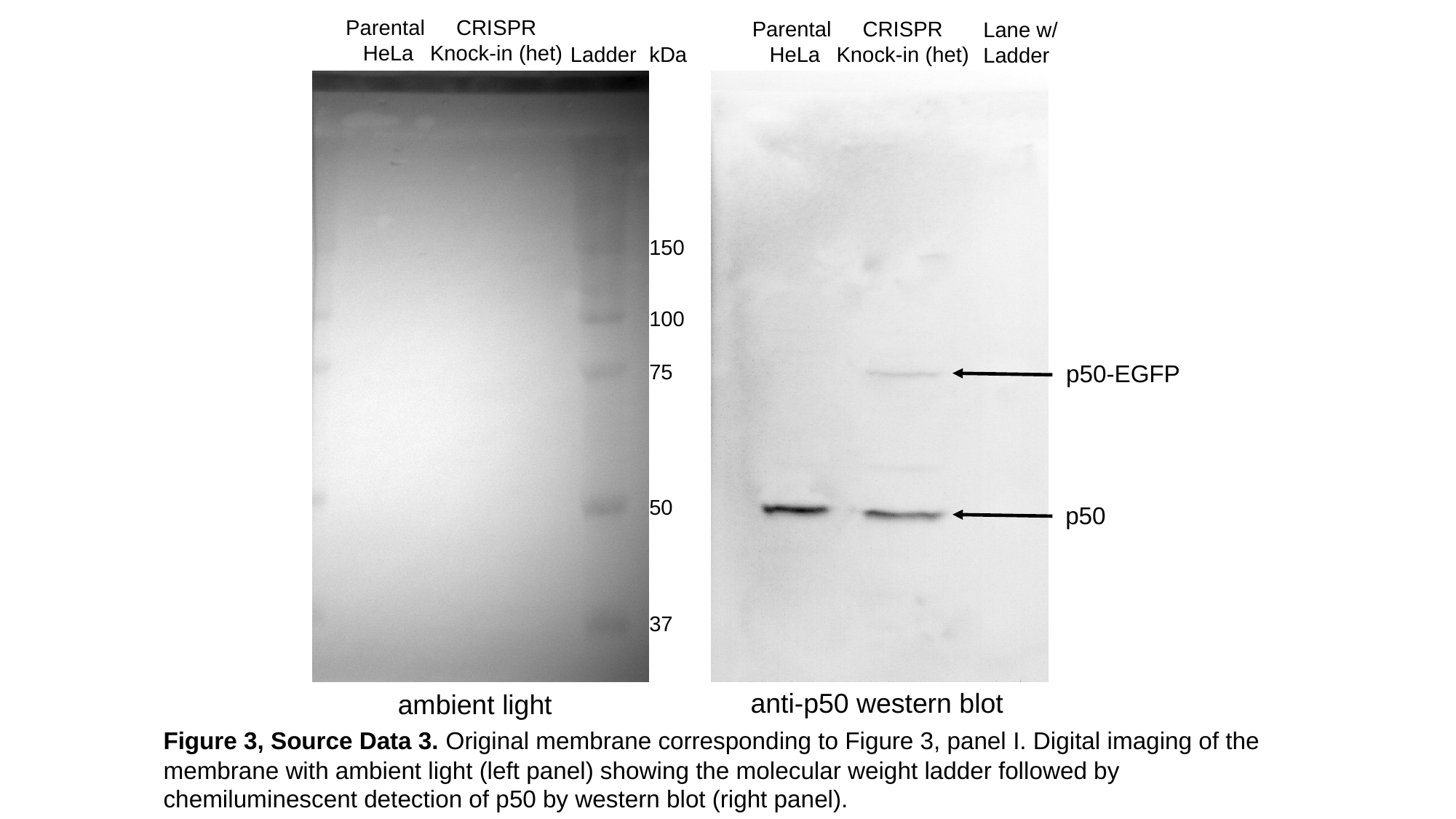

Parental
HeLa
CRISPR
Knock-in (het)
Parental
HeLa
CRISPR
Knock-in (het)
Lane w/
Ladder
Ladder
kDa
150
100
75
p50-EGFP
50
p50
37
anti-p50 western blot
ambient light
Figure 3, Source Data 3. Original membrane corresponding to Figure 3, panel I. Digital imaging of the membrane with ambient light (left panel) showing the molecular weight ladder followed by chemiluminescent detection of p50 by western blot (right panel).
